# Epidermal growth factor attenuates blood‐spinal cord barrier disruption *via* PI3K/Akt/Rac1 pathway after acute spinal cord injury

**DOI:** 10.1111/jcmm.12761

**Published:** 2016-01-15

**Authors:** Binbin Zheng, Libing Ye, Yulong Zhou, Sipin Zhu, Qingqing Wang, Hongxue Shi, Daqing Chen, Xiaojie Wei, Zhouguang Wang, Xiaokun Li, Jian Xiao, Huazi Xu, Hongyu Zhang

**Affiliations:** ^1^ Department of OrthopaedicsThe Second Affiliated HospitalWenzhou Medical UniversityWenzhouZhejiangChina; ^2^ School of Pharmaceutical SciencesKey Laboratory of Biotechnology and Pharmaceutical EngineeringWenzhou Medical UniversityWenzhouZhejiangChina; ^3^ Emergency DepartmentThe Second Affiliated HospitalWenzhou Medical UniversityWenzhouZhejiangChina; ^4^ Department of NeurosurgeryCixi People's HospitalWenzhou Medical UniversityNingboZhejiangChina

**Keywords:** blood–spinal cord barrier, epidermal growth factor, Rac1, PI3K/Akt, spinal cord injury

## Abstract

After spinal cord injury (SCI), disruption of blood–spinal cord barrier (BSCB) elicits blood cell infiltration such as neutrophils and macrophages, contributing to permanent neurological disability. Previous studies show that epidermal growth factor (EGF) produces potent neuroprotective effects in SCI models. However, little is known that whether EGF contributes to the integrity of BSCB. The present study is performed to explore the mechanism of BSCB permeability changes which are induced by EGF treatment after SCI in rats. In this study, we demonstrate that EGF administration inhibits the disruption of BSCB permeability and improves the locomotor activity in SCI model rats. Inhibition of the PI3K/Akt pathways by a specific inhibitor, LY294002, suppresses EGF‐induced Rac1 activation as well as tight junction (TJ) and adherens junction (AJ) expression. Furthermore, the protective effect of EGF on BSCB is related to the activation of Rac1 both *in vivo* and *in vitro*. Blockade of Rac1 activation with Rac1 siRNA downregulates EGF‐induced TJ and AJ proteins expression in endothelial cells. Taken together, our results indicate that EGF treatment preserves BSCB integrity and improves functional recovery after SCI *via* PI3K‐Akt‐Rac1 signalling pathway.

## Introduction

Spinal cord injury (SCI), a serious health problem that usually initiates permanent disability, leads to direct vascular damage and a cascade of events that adjust the permeability of the blood–spinal cord barrier (BSCB) 1, 2. The BSCB is the functional equality of the blood‐brain barrier (BBB), offering a specialized microenvironment for the cellular constituents of the spinal cord. The barrier function of BSCB is based on the specialized system of non‐fenestrated endothelial cells and their accessory structures, including the basement membrane, pericytes and astrocytic end feet processes, which play a protective and regulatory role for the spinal cord parenchyma 3. Previous studies show that the disruption of BSCB participates in the pathophysiological processes of SCI, for example, spinal cord oedema and secondary nerve injury. The disruption of BSCB causes inflammation and blood infiltration and then engenders neurotoxic products that compromise neuronal and synaptic functions and causes the ‘programmed death’ of glia and neurons, in result, it leads to permanent neurological deficits 4, 5. Recently, it reports that the disruption of BSCB is correlated with increased mortality after intravascular therapy, and improvements in the BSCB function can significantly diminish secondary nerve injury 6. In general, it suggests that early recovery of BSCB plays a vital role in the treatment of SCI.

Rac1, a member of small GTPases, is well‐characterized in the Rho family 7. Intracellularly, the GTP‐bound form of Rac1 performs a mutual effect on downstream effectors that operate multiple cellular processes, including membrane trafficking 8, gene transcription 9, the formation and maintenance of cell–cell junctions and the establishment of epithelial barriers 10, 11. For example, the small GTPases Rac1 regulates adherens junction (AJ) function in epithelial cells and participates in the formation of the epithelial permeability barrier in human airway epithelial cells 12, 13, 14. Daniels *et al*. also reports that the small GTPases Rac1 has effect on the regulation of endothelial permeability and tight junction (TJ) formation by using an *in vitro* BBB model 15. In addition, it is well‐established that the PI3K/Akt pathway is required for the stability of barrier function. A recent study shows that miR‐21 regulates intestinal epithelial TJ (Occludin, Claudin‐1) permeability through PTEN/PI3K/Akt signalling pathway 16. In the retina, activation of the PI3K/AKT pathway is involved in the expression of ZO1 and Occludin levels, which are synthesized by Pigment Epithelium‐Derived Factor Peptide 17. Gunduz *et al*. also reports that insulin stabilizes endothelial barrier function *via* Rac1 activation induced by PI3K/Akt 18. According to studies above, we find that PI3K/Akt and Rac1 are involved in regulating barrier permeability, however, the role of PI3K/Akt and Rac1 on BSCB after SCI is unclear.

As a widely expressed protein, epidermal growth factor (EGF) has the ability to coordinate different aspects of cell proliferation, growth, differentiation and morpho‐functional maintenance *via* a more complex signal transduction system. Epidermal growth factor is a neurotrophic factor that promotes survival and protraction of midbrain dopaminergic neurons 19, 20, 21. After SCI in rats, EGF can improve functional recovery by promoting the division, differentiation and migration of a large number of ependymal cells, including endogenous neural precursor cells and atrocities 22. Although EGF shows protective effects on SCI 23, 24, its influence on the BSCB and underlying signalling pathway after SCI remains unclear.

In this study, we demonstrate that EGF administration attenuates secondary SCI, specifically by preserving endothelial TJ and AJ; therefore it attenuates neurofunctional deficits in the rat subjected to SCI. Furthermore, EGF improves the permeability of BSCB by enhancing TJ and AJ proteins expression through activation of the PI3K/Akt/Rac1 pathway.

## Materials and methods

### Spinal cord injury

The adult female Sprague–Dawley rats (220–250 g) were obtained from the Animal Center of the Chinese Academy of Sciences. All animal experiments were conformed to the Guide for the Care and Use of Laboratory Animals from the National Institutes of Health and were approved by the Animal Care and Use Committee of Wenzhou University. All animals were housed in standard temperature conditions with 12 hrs light/dark cycle and fed with food and water. Rats were anaesthetized with 10% chloralic hydras (3.5 ml/kg, i.p.) and a laminectomy was performed at the T9 level, exposing the cord beneath without disrupting the dura. The exposed spinal cord was subjected to moderate contusion injury (150 kdyn force with no dwell time) using an Infinite Horizon Impact Device. The sham‐operated group rats underwent a T9 laminectomy without contusion injury. Postoperative care included manual urinary bladder emptying per 12 hrs until the return of bladder function and the administration of cefazolin sodium (50 mg/kg, i.p.).

### Drug treatment

Epidermal growth factor (Sigma‐Aldrich, St. Louis, MO, USA) dissolved in 0.9% NaCl (60 μg/kg) was injected subcutaneously near the back wound after SCI and treated once a day for 1 week for behavioural test or for indicated time‐points for other experiments. PI3K inhibitor LY294002 (Sigma‐Aldrich) were dissolved in 25% dimethylsulphoxide solution. A total volume of 5 μl (50 nmol/kg) solution was injected into the spinal cord *via* intrathecal injection in 5 min. For the sham‐operated group rats, they received no pharmacological treatment.

### Cell culture

Human brain microvascular endothelial cells (HBMECs) were purchased from ScienCell Research Laboratories (ScienCell Research Laboratories, San Diego, CA, USA). Cells were cultured in endothelial cell medium (ScienCell Research Laboratories) and incubated in a humidified atmosphere contain 5% CO_2_ at 37°C. Cells were pretreated for 2 hrs with EGF (100 ng/ml), EGF compound with LY294002 (20 μM). All experiments were performed in triplicate.

### Behavioural tests

Examination of functional deficits after SCI was conducted as previously described 25. The Basso, Beattie, and Bresnahan (BBB) scores were assessed in an open field scale by two blinded independent examiners at 14 days post‐operation. Briefly, the BBB locomotion rating scale scores range from 0 points (complete paralysis) to 21 points (normal locomotion). The scale was based on the natural progression of locomotion recovery in rats with thoracic SCI.

### Western blot analysis

For protein analysis *in vivo*, the protein extraction was homogenized in a modified RIPA buffer (50 mM Tris–HCl, 1% NP‐40, 20 mM DTT, 150 mM NaCl, pH = 7.4) containing protease inhibitor cocktail (10 μl/ml; GE Healthcare Biosciences, PA, Little Chalfont, UK). The complex was then centrifuged at 11,792 ***g***, and the supernatant was obtained for protein assay. For protein analysis *in vitro*, HBMECs were lysed in RIPA buffer [25 mM Tris–HCl (pH 7.6), 150 mM NaCl, 1% Nonidet P‐40, 1% sodium deoxycholate and 0.1% SDS] with protease and phosphatase inhibitors. The extracts above were quantified with bicinchoninic acid reagents (Thermo, Rockford, IL, USA). The equivalent of 50 μg protein was separated using 12% gel and then transferred onto a PVDF membrane (Bio‐Rad, Hercules, CA, USA). The membrane was blocked with 5% non‐fat milk in TBS with 0.05% Tween 20 for 1 hr, then incubated with following antibody solutions: p120‐Catenin, beta‐Catenin, Occludin, Claudin‐5, p‐Akt, Akt, Rac1, GAPDH. The membranes were washed with TBS three times and incubated with secondary antibodies for 2 hrs at room temperature. Signals were visualized using the ChemiDicTM XRS + Imaging System (Bio‐Rad), and band densities were quantified with Image J software. Results were expressed as a relative density ratio, normalized to the value of the Sham or Control group. Anti‐ p120‐Catenin, β‐Catenin, Rac1 were purchased from Abcam (Cambridge, UK), other antibodies were from Santa (Santa Cruz Biotechnology, Santa Cruz, CA, USA).

### Haematoxylin and eosin staining

The rats were anesthetized with 10% chloralic hydras (3.5 ml/kg, i.p.), then perfused with 0.9% NaCl, followed by 4% paraformaldehyde in 0.01 M PBS (pH = 7.4) at 7 days after surgery. The spinal cords from the T7–T10 level around the lesion epicentre were excised, transverse paraffin sections (5 mm thick) were mounted on poly‐l‐lysine‐coated slides for histopathological examination by haematoxylin and eosin staining.

### Immunofluorescence staining

The sections were incubated with 5% bovine serum albumin (BSA) for 1 hr at room temperature and then incubated overnight at 4°C with primary antibodies in blocking buffer (Claudin‐5, Santa Cruz Biotechnology; Occludin, Santa Cruz Biotechnology; CD31, Santa Cruz Biotechnology). Then the cords were separately incubated with secondary antibody (Alexa Fluor 488‐conjugated anti‐IgG, Abcam; Texas red‐conjugated anti‐IgG, Santa Cruz Biotechnology). The nuclei were stained with Hoechst 33258 (0.25 mg/ml) dye (Beyotime Institute of Biotechnology, Shanghai, China). For cells, grown on 14 × 14 mm microscopic glass were washed with ice‐cold PBS, fixed with 4% paraformaldehyde for 30 min., then washed with ice‐cold PBS, and blocked in 5% BSA for 1 hr. Then cells were incubated with anti‐p120‐Catennin (Abcam), anti‐beta‐Catenin (Abcam), anti‐Occludin (Santa Cruz Biotechnology), anti‐Claudin‐5 (Santa Cruz Biotechnology) diluted in 1% BSA at 4°C overnight. Cells were washed with PBS followed by incubation with Alexa Fluor 488‐conjugated anti‐IgG or Texas red‐conjugated anti‐IgG secondary antibodies for 1 hr at room temperature. After washing with PBS, the nuclei were stained with Hoechst 33258 (0.25 mg/ml) dye for 7 min., washed with PBS. At last, cells were added with Antifade Mounting Medium (Beyotime Institute of Biotechnology).

### Pull‐down assays

GTP‐bound active form of Rac1 was detected by Active Rac1 Pull‐Down and Detection Kit (Thermo Scientific, Mass, Waltham, USA) according to the manufacturer's protocol. Extracts of HBMECs were incubated at 4°C for 60 min. with GST‐human Pak1‐PBD, Bound proteins were mixed with 1 part β‐mercaptoethanol to 20 parts 2× SDS Sample Buffer and boiled for 5 min. GTP‐bound Rac1 were detected by Western blotting with the anti‐Rac1 antibody.

### Small interfering RNA

Rac1 expression was silenced by transfection of small interfering RNA (siRNA). Human brain microvascular endothelial cells were transfected with 100 pmol of Rac1 siRNA (Bioneer, Daejeon, Korea) in serum‐free medium mixed with lipofectamine 2000 (Life Technologies, Carlsbad, CA, USA) according to the manufacturer's instructions. Four hours after transfection, HBMECs were incubated in a medium containing 5% foetal bovine serum for 24 hrs.

### Evaluation of BSCB permeability

#### Evans blue dye assays

At 1 day after SCI, rats were injected with 2% Evans blue dye (EB; Sigma‐Aldrich, 2 ml/kg) solution in saline intravenously into the tail vein. Two hours after injection, rats were anesthetized with 10% chloralic hydras (3.5 ml/kg, i.p), then perfused with 0.9% normal saline. The injured spinal cord tissues of EB were weighed and immersed in *N*,*N*′‐dimethylformamide (JinSan, Wenzhou, China) at 50°C for 72 hrs. The optical density of the supernatant was examined with enzyme‐labelled meter (at an excitation wavelength of 620 nm and an emission wavelength of 680 nm). Dye in samples was determined as μg/g of tissue from a standard curve plotted using known amounts of dye 26. Rats were fixed by perfusion with 4% paraformaldehyde at 2 hrs after EB injection. The spinal cord tissues were cut into 20 μm thickness at −20°C using frozensection machine, then the sections were observed.

#### FITC‐dextran assays

At 1 day after SCI, rats were injected with 2% FITC‐dextran (MW 70 kD, 4 mg/kg; Sigma‐Aldrich) solution in PBS intravenously into the tail vein. Two hours after injection, rats were anesthetized with 10% chloralic hydras (3.5 ml/kg, i.p), then perfused with 0.9% normal saline. The injured spinal cord tissues of FITC‐dextran were weighed and homogenized in PBS, and centrifuged. The optical density of the supernatant was examined (at an excitation wavelength of 493 nm and an emission wavelength of 517 nm).

#### Paracellular permeability assay

Human brain microvascular endothelial cells were seeded on Transwell permeable supports (PET membrane 24‐well cell culture inserts with 0.4 μm pore size; Corelle; Corning Life Sciences, Corning, New York, USA) at a density of 1 × 10^5^ cells/well in 200 μl medium overnight, and subjected to oxygen‐glucose deprivation (OGD) for 10 hrs, then cells were incubated with FITC‐dextran (1 mg/ml) in medium for another 2 hrs OGD. Thereafter, FITC‐dextran passed through the Transwell (in the lower chambers) was determined by using enzyme‐labelled meter at an excitation wavelength of 493 nm and an emission wavelength of 517 nm.

### Statistical analysis

Data are expressed as the mean ± S.E.M. Statistical significance was determined with Student's *t*‐test when there were two experimental groups. For more than two groups, statistical evaluation of the data was performed with the one‐way anova test, Tukey's multiple comparison is used as a *post hoc* analysis.

## Results

### EGF improves the functional recovery and attenuates BSCB disruption after SCI

**Figure 1:**
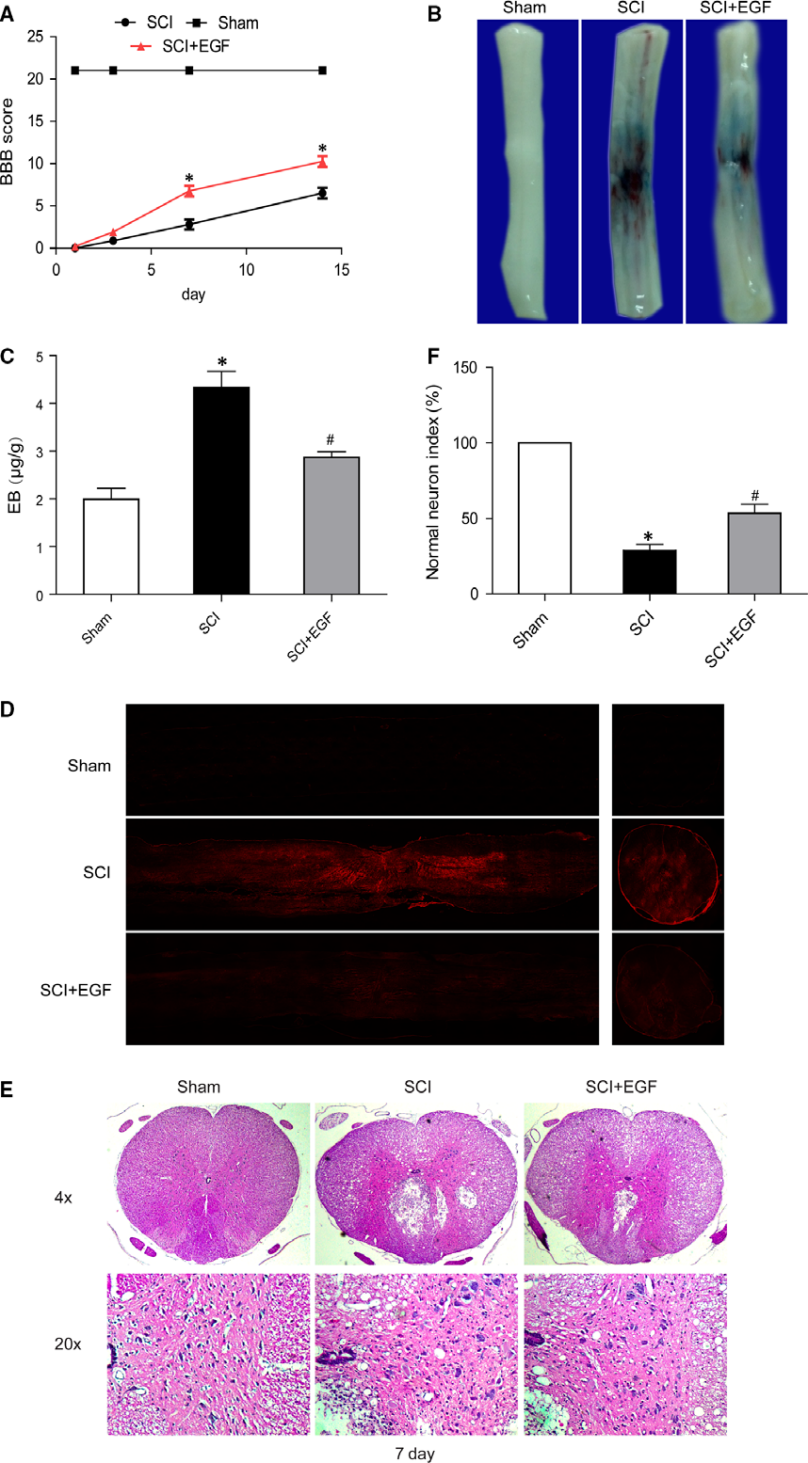
Epidermal growth factor improves the functional recovery and attenuates BSCB disruption after SCI. (**A**) The Basso, Beattie and Bresnahan (BBB) scores, *represents *P* < 0.01 *versus* the SCI group, *n* = 5. (**B**) Representative whole spinal cords show that Evan's Blue dye permeabilized into injury spinal cord at 1 day (*n* = 4/group). (**C**) Quantification of the amount of Evan's Blue at 1 day (μg/g), *represents *P* < 0.01 *versus* the Sham group, ^#^represents *P* < 0.05 *versus* the SCI group. (**D**) Representative confocal images of Evans Blue Dye extravasation at 1 day after SCI. (**E**) H and E staining at 7 day. (**F**) A quantitative analysis of normal neurons in haematoxylin and eosin staining, *represents *P* < 0.01 *versus* the Sham group, ^#^represents *P* < 0.01 *versus* the SCI group.

After SCI, rats were immediately treated with EGF and further treated once a day for 1 week. Functional recovery was then estimated using BBB scores for locomotion. As a result, EGF treatment significantly increased the locomotor activity 3–14 days after injury, compared with that observed in SCI group (Fig. 1A). Meanwhile, we examined the effect of EGF on BSCB permeability at 1 day after SCI by Evan's Blue assay. As shown in Figure 1B, the amount of Evan's Blue dye extravasation had a marked increase compared with the Sham group after SCI, which implies BSCB disruption. Furthermore, EGF treatment significantly reduced the amount of Evan's Blue dye extravasation at 1 day after SCI when compared with SCI group (Fig. 1C). The fluorescence of Evan's Blue in the injured spinal cord (at 1 day) was higher than Sham group, and EGF significantly reduced the fluorescence intensity (Fig. 1D). Traumatic SCI also triggers immediate mechanical damage. According to the experiment, Sham group showed integrated infrastructures, clear boundary between grey and white matters, morphologically normal neurons with clear profile, polygonal perikaryon and round nucleus. By contrast, in the SCI group, spinal cord has cavities, lacked of clear infrastructures. The number of normal neurons was apparently reduced. In contrast to SCI group, the EGF treatment group showed more normal neurons (Fig. 1E and F). These data indicate that EGF improves the functional recovery and inhibits the BSCB permeability after SCI.

### EGF administration prevents the loss of TJ and AJ after SCI

**Figure 2:**
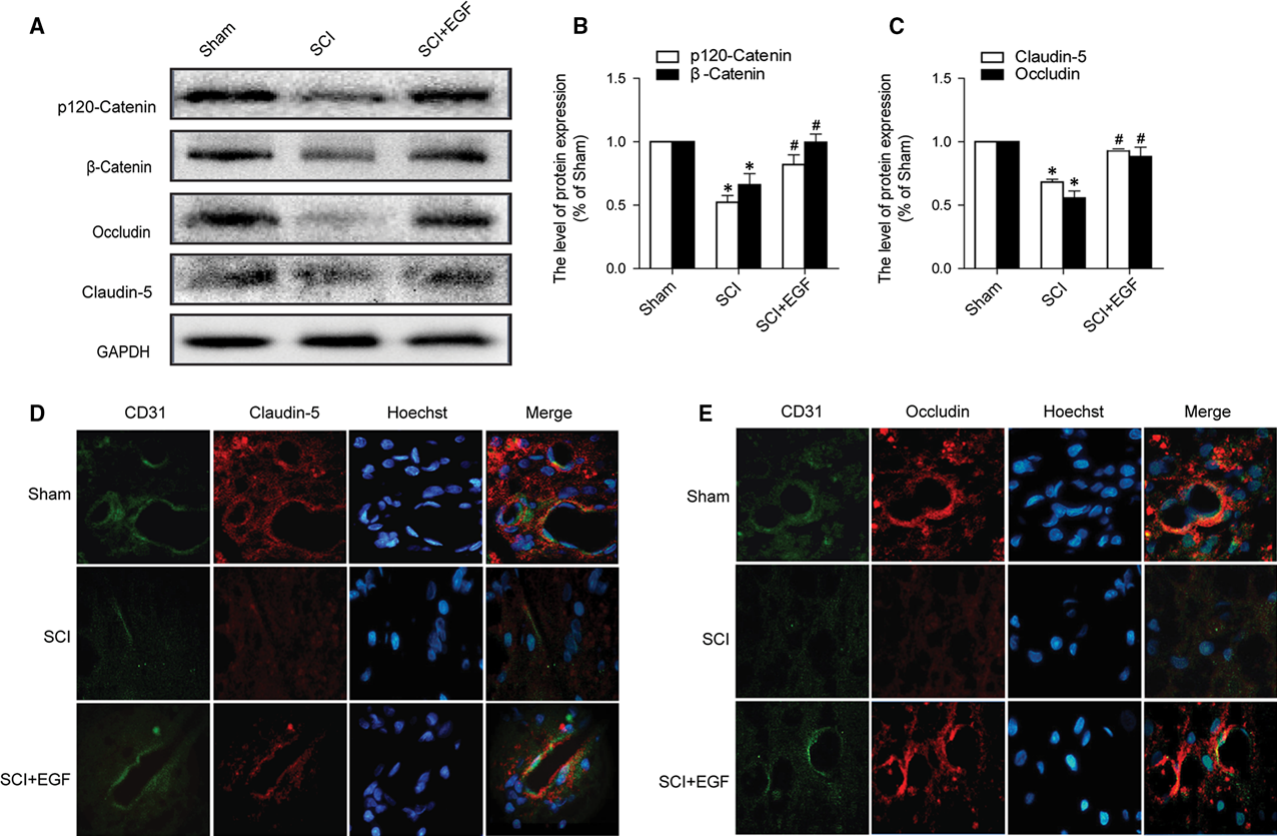
EGF administration prevents the degradation of TJ and AJ proteins after SCI. (**A**) Protein expressions of p120‐Catenin, β‐Catenin, Occludin and Claudin‐5 in the spinal cord segment at the contusion epicentre. GAPDH was used as the loading control and for band density normalization. (**B** and **C**) The optical density analysis of p120‐Catenin, β‐Catenin, Occludin and Claudin‐5 protein, *represents *P* < 0.05 *versus* the Sham group, ^#^represents *P* < 0.05 *versus* the SCI group, *n* = 5. (**D** and **E**) Double immunofluorescence shows that TJ and AJ proteins colocalize in CD31 (endothelial cell marker)‐positive blood vessels in the Sham, SCI rat and SCI rat treated with EGF groups.

It is well known that TJ and AJ in the endothelial cells of blood vessels are involved in the integrity of BBB or BSCB 3. We examined the alterations of SCI‐induced TJ and AJ proteins and the effect of EGF on these alterations by western blot. As shown in Figure 2A, the levels of TJ (Occludin, Claudin‐5) and AJ (p120‐Catenin, β‐Catenin) were decreased at 1 day after SCI. Furthermore, EGF significantly attenuated the decrease in TJ and AJ levels at 1 day after injury compared with SCI group (Fig. 2B and C). Double labelling immunofluorescence also showed that the fluorescence intensity of Occludin or Claudin‐5 and CD31 immunoreactivity was decreased after SCI compared to the Sham group, and EGF treatment attenuated the decrease in its intensity (Fig. 2D and E). These data indicate that EGF prevents BSCB disruption by inhibiting degradation of AJ and TJ proteins after SCI.

### PI3K inhibition reverses the protective effect of EGF on BSCB

**Figure 3:**
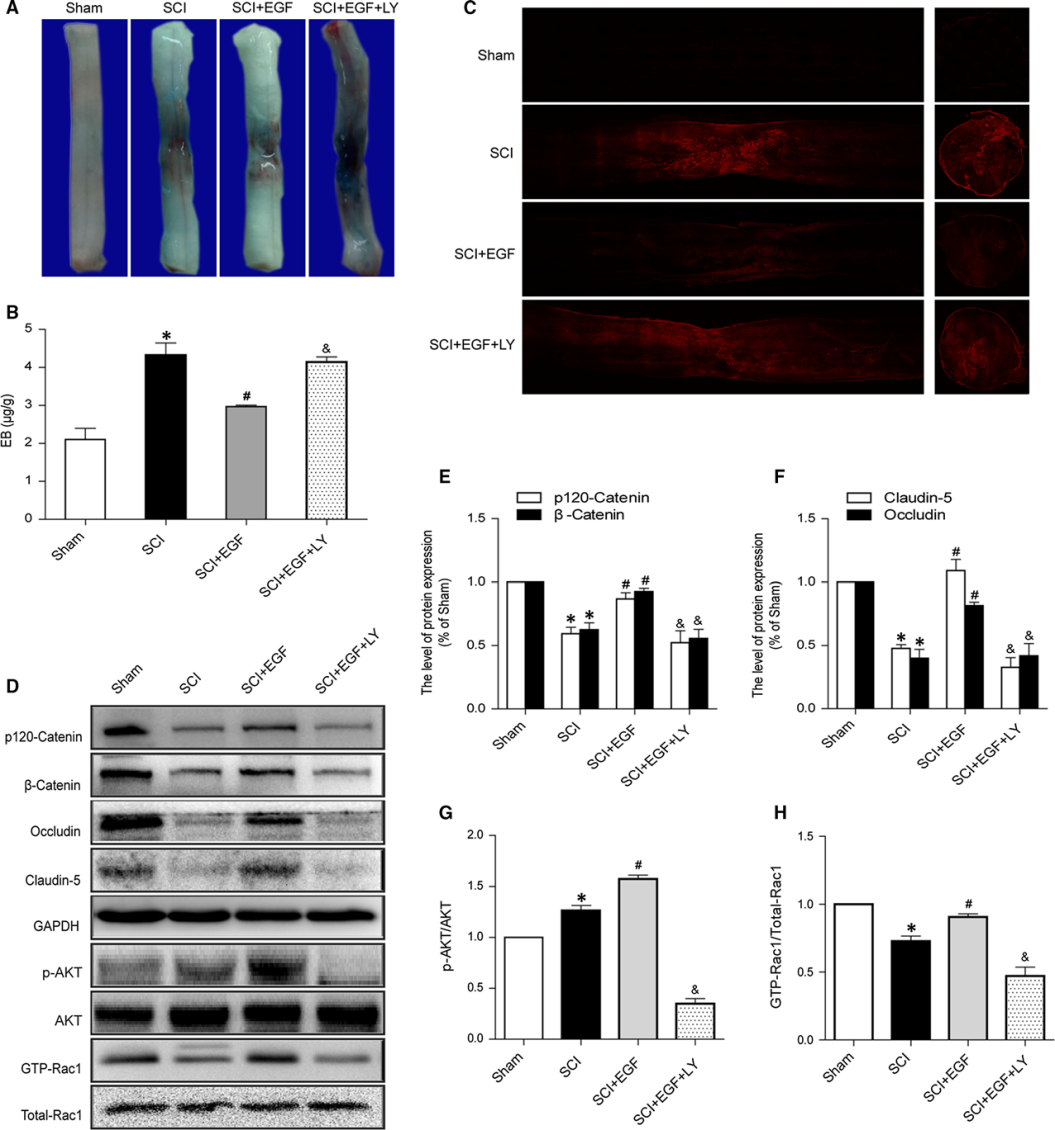
PI3K inhibitor LY294002 attenuates the protective effect of EGF on BSCB after SCI. (**A**) Representative whole spinal cords show that Evan's Blue dye permeabilized into injury spinal cord at 1 day (*n* = 4/group). (**B**) Quantification of the amount of Evan's Blue at 1 day (μg/g). (**C**) Representative confocal images of Evans Blue Dye extravasation at 1 day of Sham, SCI group, SCI rat treated with EGF group and SCI rat treated with EGF and LY294002 group. (**D**) Protein expressions of p120‐Catenin, β‐Catenin, Occludin, Claudin‐5, p‐Akt, Akt, GTP‐Rac1 and Total‐Rac1 in the spinal cord segment at the contusion epicentre. (**E** and **F**) The optical density analysis of p120‐Catenin, β‐Catenin, Occludin and Claudin‐5 protein. (**G** and **H**) The optical density analysis of p‐Akt/Akt and GTP‐Rac1/Total‐Rac1 proteins. *represents *P* < 0.05 *versus* the Sham group, ^#^represents *P* < 0.05 *versus* the SCI group, & represents *P* < 0.05 *versus* the SCI+EGF group, *n* = 5.

To evaluate whether PI3K was involved in the protection by EGF, PI3K inhibitor LY294002 was co‐administered with EGF. LY294002 alone has no toxic and devastating effect on BSCB (Fig. S1). Evans Blue dye extravasation was examined at 24 hrs after SCI. The results of the EGF+LY group were going to compare with those of the Sham, SCI, and SCI+EGF group, respectively. LY294002 co‐administration reversed the protection caused by EGF as shown by the results in the Evans Blue dye (Fig. 3A), Evans Blue extravasation assay (Fig. 3B). EGF+LY group performed significantly more dye extravasation than animals that received EGF treatment. Furthermore, the animals treated with EGF+LY had significantly increased the fluorescence intensity than EGF group in the injured spinal cord (at 1 day). Meanwhile, Western blot analyses were performed with the spinal cord of animals at 24 hrs after SCI and subsequent co‐administration of EGF and LY294002. As shown in Figure 3, EGF treatment significantly increased the protein level of p‐Akt, comparing to both the Sham and the SCI group. The PI3K inhibitor LY294002 (EGF+ LY) reversed this treatment effect (Fig. 3D and G). In addition, GTP‐Rac1/Total‐Rac1‐ratio was increased significantly in the EGF group compared to the SCI group, but decreased in the EGF+LY group (Fig. 3D and H). Meanwhile the ratios of p120‐Catenin, β‐Catenin, Occludin and Claudin‐5 were significantly decreased in the EGF+LY group (Fig. 3D–F). These data imply that PI3K inhibition (LY294002) blocks the protective effect of EGF on BSCB after SCI.

### PI3K inhibition attenuates the protective effect of EGF on junction protein in endothelial cells after oxygen‐glucose deprivation

**Figure 4:**
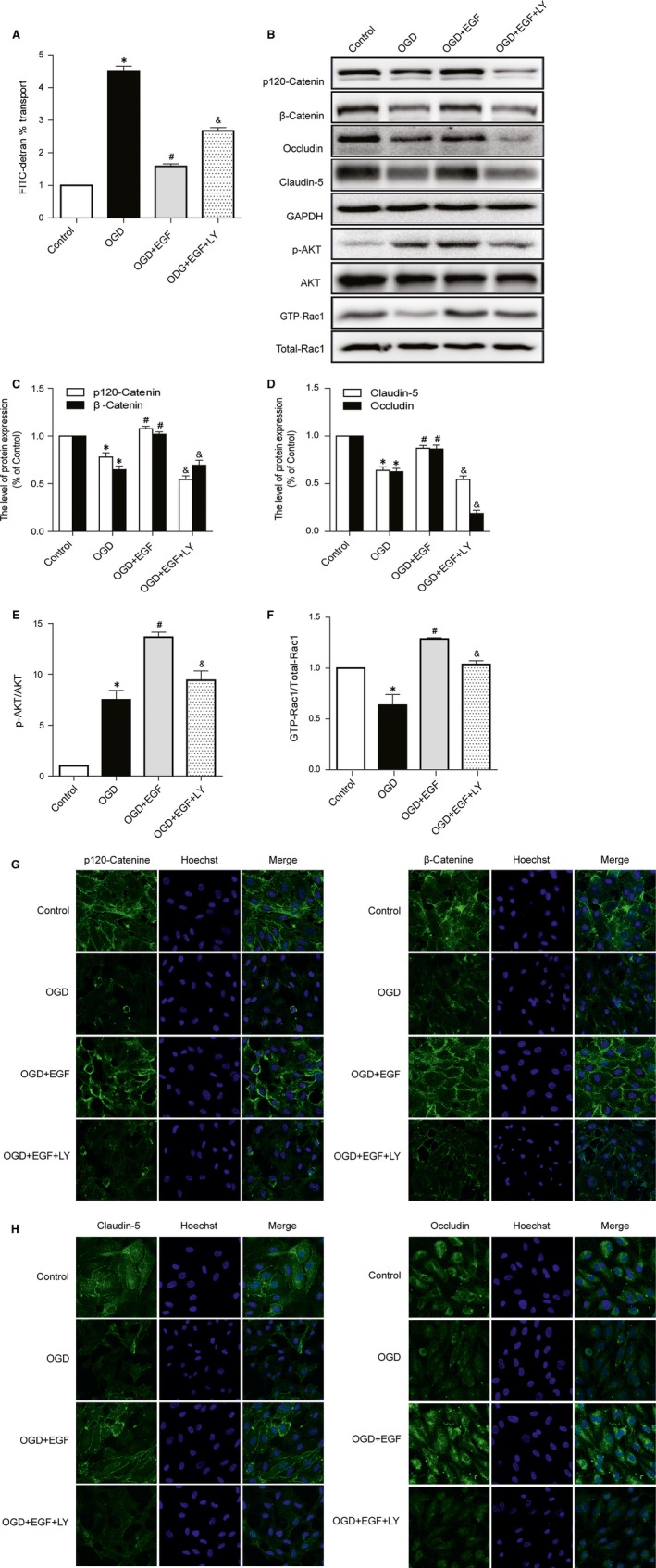
PI3K inhibitor LY294002 attenuates the protective effect that EGF inhibits the loss of TJ and AJ in Human brain microvascular endothelial cells after OGD. (**A**) Evaluation of paracellular permeability by FITC–dextran. (**B**) Protein expressions of p120‐Catenin, β‐Catenin, Occludin, Claudin‐5, p‐Akt, Akt, GTP‐Rac1 and Total‐Rac1. (**C** and **D**) The optical density analysis of p120‐Catenin, β‐Catenin, Occludin and Claudin‐5 protein. (**E** and **F**) The optical density analysis of p‐Akt/Akt and GTP‐Rac1/Total‐Rac1 proteins. (**G** and **H**) Immunofluorescence staining of TJ and AJ protein. Nucleus (blue) was labelled with Hoechst. *represents *P* < 0.05 *versus* the Control group, #represents *P* < 0.05 *versus* the OGD group, & represents *P* < 0.05 *versus* the OGD + EGF group.

To further confirm the potential mechanism of EGF mediated regulation TJ and AJ *in vitro*, we applied OGD to HBMECs. Paracellular permeability assay of FITC‐dextran were measured to evaluate whether the inhibition of PI3K would affect the permeability of BSCB. LY294002 alone has no toxic effect in endothelial cells (Fig. S1). As shown in Figure 4A, the permeability values to FITC–dextran were reduced in EGF group compared with the OGD group. However, LY294002 co‐administration increased the permeability. Western blot analyses were performed with the HBMECs at 12 hrs after OGD. As shown in Figure 4, the levels of TJ (Occludin, Claudin‐5) and AJ (p120‐Catenin, β‐Catenin) decreased at 12 hrs after OGD. However, the decrease in TJ (Occludin, Claudin‐5) and AJ (p120‐Catenin, β‐Catenin) level after OGD was notably reversed by EGF treatment. The group treated with EGF significantly increased the protein level of p‐Akt, comparing to both the Control and the OGD group. In addition, GTP‐Rac1/Total‐Rac1 ratio was significantly increased in the EGF group as compared to the OGD group. However, LY294002 administration evidently decreased the protein level of p‐Akt as well as the GTP‐Rac1/Total‐Rac1 ratio, the levels of TJ (Occludin, Claudin‐5) and AJ (p120‐Catenin, β‐Catenin) were also significantly decreased in the EGF+LY group. Cell immunofluorescence also showed that the fluorescence intensity of TJ (Occludin, Claudin‐5) and AJ (p120‐Catenin, β‐Catenin) decreased after OGD as compared to the Control group, and EGF treatment attenuated the decrease in its intensity. However, LY294002 administration significantly decreased the fluorescence intensity (Fig. 4G and H). These data indicate that PI3K inhibition reverses the protective effect of EGF on TJ and AJ in endothelial cell after OGD.

### Rac1 activation is required for EGF‐induced TJ and AJ proteins expression

**Figure 5:**
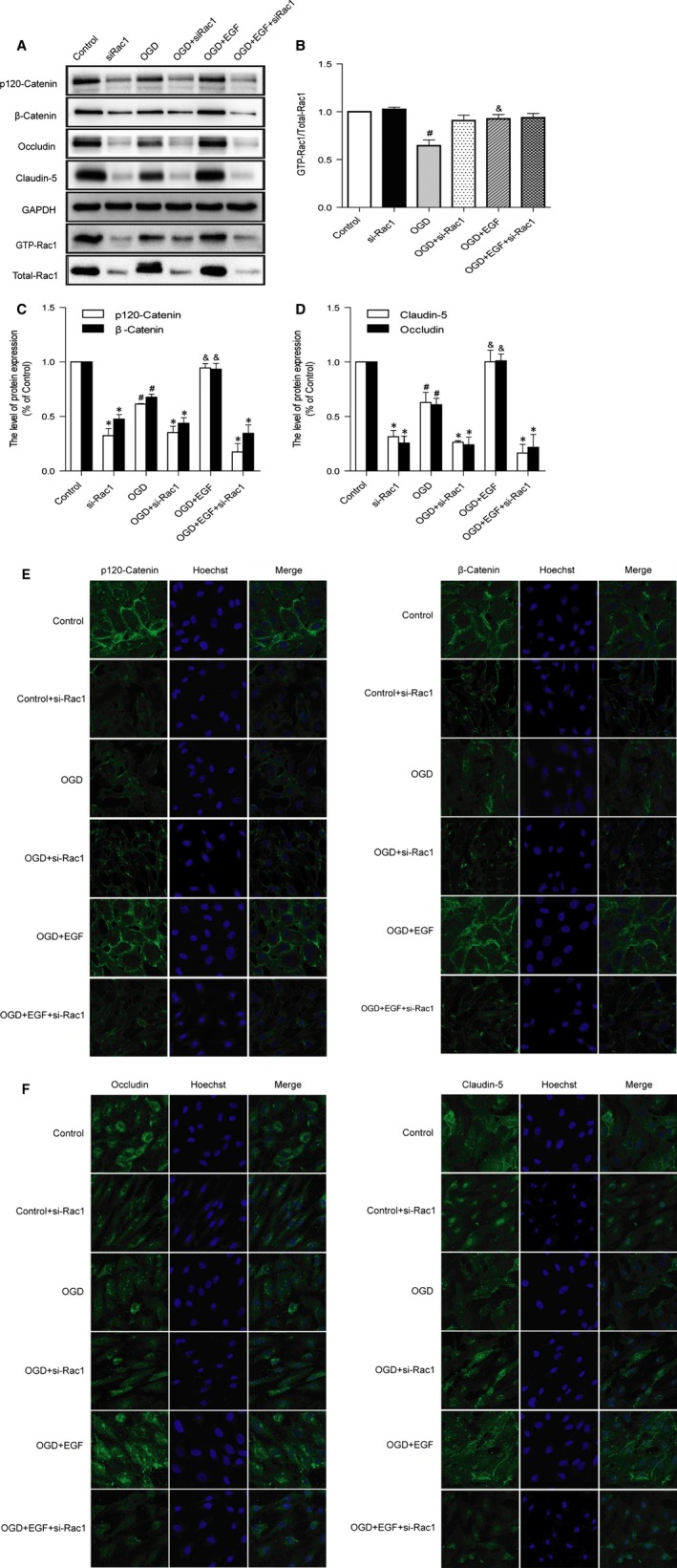
Rac1 activation is required for EGF‐induced TJ and AJ proteins expression. (**A**) Rac1 silencing inhibits the protein expressions of p120‐Catenin, β‐Catenin, Occludin, Claudin‐5. (**B**–**D**) The optical density analysis of p120‐Catenin, β‐Catenin, Occludin, Claudin‐5 and GTP‐Rac1/Total‐Rac1 protein. *represents *P* < 0.05 *versus* the corresponding non‐si‐Rac1 group, ^#^represents *P* < 0.05 *versus* the Control group, & represents *P* < 0.05 *versus* the OGD group. (**E** and **F**) Immunofluorescence staining of TJ and AJ proteins. Nucleus (blue) was labelled with Hoechst.

To evaluate whether Rac1 was involved in the protection by EGF on TJ and AJ, we administrated Rac1 siRNA in endothelial cells. *In vitro* delivery of siRNA targeting Rac1 inhibited Rac1 expression and activity of Rac1 (Fig. 5A and B). Meantime, the expression of AJ and TJ proteins were much lower in Rac1 silence than Rac1 activation (Fig. 5A, C and D). In addition, the protective effect of EGF on TJ and AJ also significantly inhibited by silencing Rac1. Cell immunofluorescence also showed that the fluorescence intensity of TJ and AJ was decreased after silencing Rac1 (Fig. 5E and F). These data imply that Rac1 activation is necessary for the protective effect of EGF on TJ and AJ in endothelial cells.

## Discussion

In this study, we displayed that EGF administration attenuated neurofunctional deficits by preventing BSCB disruption in rats after SCI. EGF treatment induced phosphorylation and activation of Akt, and Rac1 (GTP‐Rac1). Ultimately, EGF treatment led to the increasing of TJ (Occludin, Claudin‐5) and AJ (P120‐Catenin, β‐Catenin) at 24 hrs after SCI. However, co‐administration of PI3K inhibitor LY294002 and EGF reversed the protective effect of EGF on BSCB, implying that the observed protection of BSCB was mediated by EGF‐induced activation of the PI3K/Akt/Rac1 signalling pathway. Here, we present the mechanism of EGF that affects the BSCB integrity after injury.

Blood–spinal cord barrier integrity plays a pivotal role in maintaining the normal functions of spinal cord. Disruption of BSCB integrity happens owing to numerous pathological conditions such as amyotrophic lateral sclerosis and SCI 3, which leads to an increasing of vascular permeability. It has been demonstrated that grow factors like NGF, FGF‐2 improve functional recovery after SCI 25, 27. However, the study of grow factors on BSCB is little done. Epidermal growth factor is a key growth factor involved in a wide range of physiological and pathologic processes including angiogenesis and wound healing 28. It plays an essential role in migration, proliferation and differentiation of endothelial cells. In the present study, we demonstrated the protective effect and molecular mechanism of EGF on BSCB *in vivo* and *in vitro*. Previous report shows that activation of EGFR and PI3K/Akt increases transepithelial resistance and improves epithelial paracellular permeability in canine kidney II cells 29. Epidermal growth factor administration promotes intestinal proliferation and improves mucosal barrier by decreasing gut permeability, recruiting TJ proteins and modulating inflammation responses after ischaemia/reperfusion injury in rats 30. In addition, EGF increases the expression of claudin‐3 protein mediated by the ERK1/2 and PI3K‐Akt pathways in HT‐29 cells 31. It improves epithelial paracellular permeability by regulating claudin‐2 and ‐4 expression through Src and STAT3 in MDCK cells 32. However, few studies report concerning the role of EGF in the regulation of BSCB permeability. A previous study demonstrates that EGF promotes neurovascular protection by targeting early BBB disruption after ischaemia–reperfusion in rats 33. As shown in this study, EGF attenuated the disruption of TJ and AJ, thereby reduced the permeability of BSCB both *in vitro* and *in vivo*.

However, the underlying mechanism between EGF and permeability barrier function is still not fully understood. As is known to all, PI3K/Akt pathway can be triggered by EGF or other trophic factors, such as bFGF and NGF 34, 35, 36. The previous study shows that bFGF improves recovery from SCI by activating PI3K/Akt pathway 37. Meanwhile PI3K/Akt is a major signalling pathway in regulating angiogenesis *in vivo* and *in vitro* 38, 39. It has been reported that PI3K/Akt signalling pathway promotes the VEGF expression as well as makes an increase in the development of choroidal neovascularization 40. Moreover, the PI3K/Akt signalling pathway regulates paracellular claudin‐5 expression in mouse brain endothelial cells and improves intestinal epithelial permeability by regulating Occludin and Claudin‐1 in Caco‐2 cells 16, 41. FGFs preserve BBB integrity after experimental intracerebral haemorrhage in mice involves PI3K/Akt pathway 42. Activation of PI3K/Akt pathway attenuates BBB disruption following neonatal hypoxia‐ischaemia in rats 43. Above research indicate that PI3K/Akt pathway is essential for mediating barrier permeability under a variety of circumstances. Compared to these findings, our results revealed that EGF elevated the level of Akt activity *in vivo* and *in vitro*. Furthermore, the specific chemical inhibitor (LY294002) for this protein causes the reduction in TJ and AJ, thereby accelerating the disruption of the permeability of BSCB. Therefore, it may be reasonable to speculate that EGF enhances the expression of TJ and AJ proteins mediated through PI3K/Akt signalling pathway.

Rac1, a member of small GTPases in the Rho family, regulates intercellular junctions and the cytoskeleton. It is a major signalling component that is known to regulate various pathways downstream of the EGFR 44. Rac1 activation promotes angiogenesis of various types of vascular endothelial cell, meanwhile it is also known to regulate the formation and function of AJ and TJ. For instance, Rac1 signalling protects AJ (E‐cadherin and β‐Catenin) from disassembly during ATP depletion in MDCK cells 13. In pulmonary endothelial barrier, Rac1 activation is necessary for VE‐cadherin redistribution 45. Therefore, we explored the role of Rac1 in BSCB. As is known to all, Rac1 appears as a vital effector to stabilize endothelial E‐cadherin and β‐Catenin, and promote AJ formation through stimulating lamellipodia formation 46, 47. Inhibition of Rac1 activation disrupts AJ, leading to increase in endothelial permeability in mesenteric microvessels and cultured cells 48, 49. The above results of study reveal that Rac1 activation is essential for mediating barrier permeability. In the present study, it was found that EGF‐induced Rac1 activation *in vivo* and *in vitro*. While preventing Rac1 activity by Rac1 siRNA increased the TJ and AJ reduction under OGD, reversed the protection of EGF on BSCB in HBMECs, suggesting that Rac1 activity was responsible for regulating the expression of TJ and AJ proteins. Previous study shows that Rac1 acts as the downstream of PI3K/Akt 50, 51, 52. Rac1 activation is stimulated by hypoxia in breast cancer cells *via* PI3K/Akt signalling 53. PI3K is involved in Rac1 activation in cardiomyocytes during LPS stimulation 54. Moreover, NGF accelerates cutaneous wound healing by promoting the migration of dermal fibroblasts *via* the PI3K/Akt‐Rac1‐JNK and ERK pathways 55. In this study, we examined that PI3K inhibitor (LY294002) caused the reduction of EGF‐induced Rac1 activity *in vitro and in vivo*, thus reversed the protection of EGF on BSCB integrity. Meanwhile, silencing the expression of Rac1 by Rac1 siRNA also prevented the protective effect of EGF on BSCB integrity. Therefore, it may be reasonable to believe that EGF‐induced activation of Rac1 and enhanced the expression of TJ and AJ proteins mediated through PI3K/Akt signalling pathway.

In conclusion, this study examines the protective effect of EGF on BSCB integrity after SCI. Our study shows that EGF attenuates BSCB disruption and improves functional recovery after SCI *via* the PI3K/Akt/Rac1 signalling pathway. These results indicate that the PI3K/Akt/Rac1 signalling pathway is an important signalling cascade involved in BSCB integrity. Our results suggest that EGF may provide potential therapeutic interventions for preventing BSCB disruption after SCI.

## Conflicts of interest

The authors confirm that the content of this article has no conflicts of interest.

## Supporting information


**Figure S1** LY294002 doesn't have a toxic and devastating effect by itself in the *in vivo* and in the *in vitro* treatments. (**A**) Representative whole spinal cords show that Evan's Blue dye permeabilized into injury spinal cord at 1 day (*n* = 4/group). (**B**) Quantification of the amount of Evan's Blue at 1 day (μg/g). (**C**) Protein expressions of p120‐Catenin, β‐Catenin, Occludin, Claudin‐5 in the spinal cord segment at the contusion epicentre. (**D** and **E**) The optical density analysis of p120‐Catenin, β‐Catenin, Occludin and Claudin‐5 protein. (**F**) Protein expressions of p120‐Catenin, β‐Catenin, Occludin, Claudin‐5 in endothelial cells. (**G** and **H**) The optical density analysis of p120‐Catenin, β‐Catenin, Occludin and Claudin‐5 protein. *represents *P* > 0.05 *versus* the Sham group, ^#^represents *P* > 0.05 *versus* the SCI group.

## References

[1] Whetstone WD , Hsu JY , Eisenberg M , *et al* Blood‐spinal cord barrier after spinal cord injury: relation to revascularization and wound healing. J Neurosci Res. 2003; 74: 227–39. 14515352 10.1002/jnr.10759 PMC2837839

[2] Muresanu DF , Sharma A , Lafuente JV , *et al* Nanowired delivery of growth hormone attenuates pathophysiology of spinal cord injury and enhances insulin‐like growth factor‐1 concentration in the plasma and the spinal cord. Mol Neurobiol. 2015; 52: 837–45. 26126514 10.1007/s12035-015-9298-8

[3] Bartanusz V , Jezova D , Alajajian B , *et al* The blood‐spinal cord barrier: morphology and clinical implications. Ann Neurol. 2011; 70: 194–206. 21674586 10.1002/ana.22421

[4] Hawkins BT , Davis TP . The blood‐brain barrier/neurovascular unit in health and disease. Pharmacol Rev. 2005; 57: 173–85. 15914466 10.1124/pr.57.2.4

[5] Zlokovic BV . The blood‐brain barrier in health and chronic neurodegenerative disorders. Neuron. 2008; 57: 178–201. 18215617 10.1016/j.neuron.2008.01.003

[6] Winkler EA , Sengillo JD , Sagare AP , *et al* Blood‐spinal cord barrier disruption contributes to early motor‐neuron degeneration in ALS‐model mice. Proc Natl Acad Sci USA. 2014; 111: E1035–42. 24591593 10.1073/pnas.1401595111 PMC3964055

[7] Hakoshima T , Shimizu T , Maesaki R . Structural basis of the Rho GTPase signaling. J Biochem. 2003; 134: 327–31. 14561717 10.1093/jb/mvg149

[8] McDonald P , Veluthakal R , Kaur H , *et al* Biologically active lipids promote trafficking and membrane association of Rac1 in insulin‐secreting INS 832/13 cells. Am J Physiol Cell Physiol. 2007; 292: C1216–20. 17035298 10.1152/ajpcell.00467.2006

[9] Barros P , Jordan P , Matos P . Rac1 signaling modulates BCL‐6‐mediated repression of gene transcription. Mol Cell Biol. 2009; 29: 4156–66. 19487462 10.1128/MCB.01813-08 PMC2715802

[10] Yamada S , Nelson WJ . Localized zones of Rho and Rac activities drive initiation and expansion of epithelial cell‐cell adhesion. J Cell Biol. 2007; 178: 517–27. 17646397 10.1083/jcb.200701058 PMC2064836

[11] Jou TS , Schneeberger EE , Nelson WJ . Structural and functional regulation of tight junctions by RhoA and Rac1 small GTPases. J Cell Biol. 1998; 142: 101–15. 9660866 10.1083/jcb.142.1.101 PMC2133025

[12] Ebnet K , Suzuki A , Horikoshi Y , *et al* The cell polarity protein ASIP/PAR‐3 directly associates with junctional adhesion molecule (JAM). EMBO J. 2001; 20: 3738–48. 11447115 10.1093/emboj/20.14.3738 PMC125258

[13] Gopalakrishnan S , Dunn KW , Marrs JA . Rac1, but not RhoA, signaling protects epithelial adherens junction assembly during ATP depletion. Am J Physiol Cell Physiol. 2002; 283: C261–72. 12055095 10.1152/ajpcell.00604.2001

[14] Terakado M , Gon Y , Sekiyama A , *et al* The Rac1/JNK pathway is critical for EGFR‐dependent barrier formation in human airway epithelial cells. Am J Physiol Lung Cell Mol Physiol. 2011; 300: L56–63. 21036915 10.1152/ajplung.00159.2010

[15] Daniels BP , Holman DW , Cruz Orengo L , *et al* Viral pathogen‐associated molecular patterns regulate blood‐brain barrier integrity *via* competing innate cytokine signals. MBio. 2014; 5: e01476–14. 25161189 10.1128/mBio.01476-14 PMC4173776

[16] Zhang L , Shen J , Cheng J , *et al* MicroRNA‐21 regulates intestinal epithelial tight junction permeability. Cell Biochem Funct. 2015; 33: 235–40. 25997617 10.1002/cbf.3109

[17] Liu Y , Leo LF , McGregor C , *et al* Pigment epithelium‐derived factor (PEDF) peptide eye drops reduce inflammation, cell death and vascular leakage in diabetic retinopathy in Ins2(Akita) mice. Mol Med. 2012; 18: 1387–401. 23019073 10.2119/molmed.2012.00008 PMC3533643

[18] Gunduz D , Thom J , Hussain I , *et al* Insulin stabilizes microvascular endothelial barrier function *via* phosphatidylinositol 3‐kinase/Akt‐mediated Rac1 activation. Arterioscler Thromb Vasc Biol. 2010; 30: 1237–45. 20339116 10.1161/ATVBAHA.110.203901

[19] Iwakura Y , Piao YS , Mizuno M , *et al* Influences of dopaminergic lesion on epidermal growth factor‐ErbB signals in Parkinson's disease and its model: neurotrophic implication in nigrostriatal neurons. J Neurochem. 2005; 93: 974–83. 15857400 10.1111/j.1471-4159.2005.03073.x

[20] Birecree E , King LE Jr , Nanney LB . Epidermal growth factor and its receptor in the developing human nervous system. Brain Res Dev Brain Res. 1991; 60: 145–54. 1893564 10.1016/0165-3806(91)90043-i

[21] Abe K , Saito H . Epidermal growth factor selectively enhances NMDA receptor‐mediated increase of intracellular Ca^2+^ concentration in rat hippocampal neurons. Brain Res. 1992; 587: 102–8. 1356059 10.1016/0006-8993(92)91433-f

[22] Kojima A , Tator CH . Intrathecal administration of epidermal growth factor and fibroblast growth factor 2 promotes ependymal proliferation and functional recovery after spinal cord injury in adult rats. J Neurotrauma. 2002; 19: 223–38. 11893024 10.1089/08977150252806974

[23] Jimenez Hamann MC , Tator CH , Shoichet MS . Injectable intrathecal delivery system for localized administration of EGF and FGF‐2 to the injured rat spinal cord. Exp Neurol. 2005; 194: 106–19. 15899248 10.1016/j.expneurol.2005.01.030

[24] Karimi Abdolrezaee S , Schut D , Wang J , *et al* Chondroitinase and growth factors enhance activation and oligodendrocyte differentiation of endogenous neural precursor cells after spinal cord injury. PLoS ONE. 2012; 7: e37589. 22629425 10.1371/journal.pone.0037589 PMC3358255

[25] Zhang HY , Wang ZG , Wu FZ , *et al* Regulation of autophagy and ubiquitinated protein accumulation by bFGF promotes functional recovery and neural protection in a rat model of spinal cord injury. Mol Neurobiol. 2013; 48: 452–64. 23516099 10.1007/s12035-013-8432-8

[26] Chen X , Lan X , Roche I , *et al* Caffeine protects against MPTP‐induced blood‐brain barrier dysfunction in mouse striatum. J Neurochem. 2008; 107: 1147–57. 18808450 10.1111/j.1471-4159.2008.05697.x PMC3692355

[27] Zhu SP , Wang ZG , Zhao YZ , *et al* Gelatin Nanostructured Lipid Carriers Incorporating Nerve Growth Factor Inhibit Endoplasmic Reticulum Stress‐Induced Apoptosis and Improve Recovery in Spinal Cord Injury. Mol Neurobiol. 2015; DOI:10.1007/s12035‐015‐9372‐2. 10.1007/s12035-015-9372-2 26232067

[28] Jin E , Kim JM , Kim SW . Priming of mononuclear cells with a combination of growth factors enhances wound healing *via* high angiogenic and engraftment capabilities. J Cell Mol Med. 2013; 17: 1644–51. 24118840 10.1111/jcmm.12152 PMC3914645

[29] Singh AB , Sugimoto K , Dhawan P , *et al* Juxtacrine activation of EGFR regulates claudin expression and increases transepithelial resistance. Am J Physiol Cell Physiol. 2007; 293: C1660–8. 17855771 10.1152/ajpcell.00274.2007

[30] Geng Y , Li J , Wang F , *et al* Epidermal growth factor promotes proliferation and improves restoration after intestinal ischemia‐reperfusion injury in rats. Inflammation. 2013; 36: 670–9. 23397076 10.1007/s10753-012-9591-x

[31] De Souza WF , Fortunato Miranda N , Robbs BK , *et al* Claudin‐3 overexpression increases the malignant potential of colorectal cancer cells: roles of ERK1/2 and PI3K‐Akt as modulators of EGFR signaling. PLoS ONE. 2013; 8: e74994. 24069372 10.1371/journal.pone.0074994 PMC3777902

[32] Garcia Hernandez V , Flores Maldonado C , Rincon Heredia R , *et al* EGF regulates claudin‐2 and ‐4 expression through Src and STAT3 in MDCK cells. J Cell Physiol. 2015; 230: 105–15. 24909426 10.1002/jcp.24687

[33] Pillai DR , Shanbhag NC , Dittmar MS , *et al* Neurovascular protection by targeting early blood‐brain barrier disruption with neurotrophic factors after ischemia‐reperfusion in rats*. J Cereb Blood Flow Metab. 2013; 33: 557–66. 23299242 10.1038/jcbfm.2012.201 PMC3618392

[34] Reichardt LF , Mobley WC . Going the distance, or not, with neurotrophin signals. Cell. 2004; 118: 141–3. 15260984 10.1016/j.cell.2004.07.008

[35] Itoh N , Ornitz DM . Fibroblast growth factors: from molecular evolution to roles in development, metabolism and disease. J Biochem. 2011; 149: 121–30. 20940169 10.1093/jb/mvq121 PMC3106964

[36] Cao L , Zhang L , Chen S , *et al* BDNF‐mediated migration of cardiac microvascular endothelial cells is impaired during ageing. J Cell Mol Med. 2012; 16: 3105–15. 22925160 10.1111/j.1582-4934.2012.01621.x PMC4393738

[37] Zhang HY , Zhang X , Wang ZG , *et al* Exogenous basic fibroblast growth factor inhibits ER stress‐induced apoptosis and improves recovery from spinal cord injury. CNS Neurosci Ther. 2013; 19: 20–9. 23082997 10.1111/cns.12013 PMC6493620

[38] Chung BH , Kim JD , Kim CK , *et al* Icariin stimulates angiogenesis by activating the MEK/ERK‐ and PI3K/Akt/eNOS‐dependent signal pathways in human endothelial cells. Biochem Biophys Res Commun. 2008; 376: 404–8. 18789310 10.1016/j.bbrc.2008.09.001

[39] Chung BH , Cho YL , Kim JD , *et al* Promotion of direct angiogenesis *in vitro* and *in vivo* by Puerariae flos extract *via* activation of MEK/ERK‐, PI3K/Akt/eNOS‐, and Src/FAK‐dependent pathways. Phytother Res. 2010; 24: 934–40. 19960515 10.1002/ptr.3063

[40] Yang XM , Wang YS , Zhang J , *et al* Role of PI3K/Akt and MEK/ERK in mediating hypoxia‐induced expression of HIF‐1alpha and VEGF in laser‐induced rat choroidal neovascularization. Invest Ophthalmol Vis Sci. 2009; 50: 1873–9. 19098317 10.1167/iovs.08-2591

[41] Camire RB , Beaulac HJ , Brule SA , *et al* Biphasic modulation of paracellular claudin‐5 expression in mouse brain endothelial cells is mediated through the phosphoinositide‐3‐kinase/AKT pathway. J Pharmacol Exp Ther. 2014; 351: 654–62. 25281324 10.1124/jpet.114.218339 PMC4244583

[42] Huang B , Krafft PR , Ma Q , *et al* Fibroblast growth factors preserve blood‐brain barrier integrity through RhoA inhibition after intracerebral hemorrhage in mice. Neurobiol Dis. 2012; 46: 204–14. 22300708 10.1016/j.nbd.2012.01.008 PMC3299916

[43] Li L , McBride DW , Doycheva D , *et al* G‐CSF attenuates neuroinflammation and stabilizes the blood‐brain barrier *via* the PI3K/Akt/GSK‐3beta signaling pathway following neonatal hypoxia‐ischemia in rats. Exp Neurol. 2015; 272: 135–44. 25585014 10.1016/j.expneurol.2014.12.020 PMC4499024

[44] Wu R , Coniglio SJ , Chan A , *et al* Up‐regulation of Rac1 by epidermal growth factor mediates COX‐2 expression in recurrent respiratory papillomas. Mol Med. 2007; 13: 143–50. 17592548 10.2119/2007-00005.Wu PMC1892765

[45] Wang L , Bittman R , Garcia JG , *et al* Junctional complex and focal adhesion rearrangement mediates pulmonary endothelial barrier enhancement by FTY720 S‐phosphonate. Microvasc Res. 2015; 99: 102–9. 25862132 10.1016/j.mvr.2015.03.007 PMC5032626

[46] Mehta D , Konstantoulaki M , Ahmmed GU , *et al* Sphingosine 1‐phosphate‐induced mobilization of intracellular Ca^2+^ mediates rac activation and adherens junction assembly in endothelial cells. J Biol Chem. 2005; 280: 17320–8. 15728185 10.1074/jbc.M411674200

[47] Komarova Y , Malik AB . Regulation of endothelial permeability *via* paracellular and transcellular transport pathways. Annu Rev Physiol. 2010; 72: 463–93. 20148685 10.1146/annurev-physiol-021909-135833

[48] Wojciak Stothard B , Potempa S , Eichholtz T , *et al* Rho and Rac but not Cdc42 regulate endothelial cell permeability. J Cell Sci. 2001; 114: 1343–55. 11257000 10.1242/jcs.114.7.1343

[49] Waschke J , Baumgartner W , Adamson RH , *et al* Requirement of Rac activity for maintenance of capillary endothelial barrier properties. Am J Physiol Heart Circ Physiol. 2004; 286: H394–401. 14512275 10.1152/ajpheart.00221.2003

[50] Petpiroon N , Suktap C , Pongsamart S , *et al* Kaempferol‐3‐O‐rutinoside from Afgekia mahidoliae promotes keratinocyte migration through FAK and Rac1 activation. J Nat Med. 2015; 69: 340–8. 25783411 10.1007/s11418-015-0899-3

[51] Zhu G , Fan Z , Ding M , *et al* An EGFR/PI3K/AKT axis promotes accumulation of the Rac1‐GEF Tiam1 that is critical in EGFR‐driven tumorigenesis. Oncogene. 2015; 34: 5971–82. 25746002 10.1038/onc.2015.45

[52] Madhyastha HK , Radha KS , Nakajima Y , *et al* uPA dependent and independent mechanisms of wound healing by C‐phycocyanin. J Cell Mol Med. 2008; 12: 2691–703. 18266963 10.1111/j.1582-4934.2008.00272.x PMC3828884

[53] Du J , Xu R , Hu Z , *et al* PI3K and ERK‐induced Rac1 activation mediates hypoxia‐induced HIF‐1alpha expression in MCF‐7 breast cancer cells. PLoS ONE. 2011; 6: e25213. 21980400 10.1371/journal.pone.0025213 PMC3181265

[54] Zhang T , Lu X , Beier F , *et al* Rac1 activation induces tumour necrosis factor‐alpha expression and cardiac dysfunction in endotoxemia. J Cell Mol Med. 2011; 15: 1109–21. 20518848 10.1111/j.1582-4934.2010.01095.x PMC3822624

[55] Chen JC , Lin BB , Hu HW , *et al* NGF accelerates cutaneous wound healing by promoting the migration of dermal fibroblasts *via* the PI3K/Akt‐Rac1‐JNK and ERK pathways. Biomed Res Int. 2014; 2014: 547187. 25006578 10.1155/2014/547187 PMC4055427

