# Supplementary figures and images for: Epidermal growth factor attenuates blood‐spinal cord barrier disruption via PI3K/Akt/Rac1 pathway after acute spinal cord injury
Source: J Cell Mol Med. 2016 Jan 15;20(6):1062–75. doi: 10.1111/jcmm.12761 (PMC4882989; doi:10.1111/jcmm.12761)

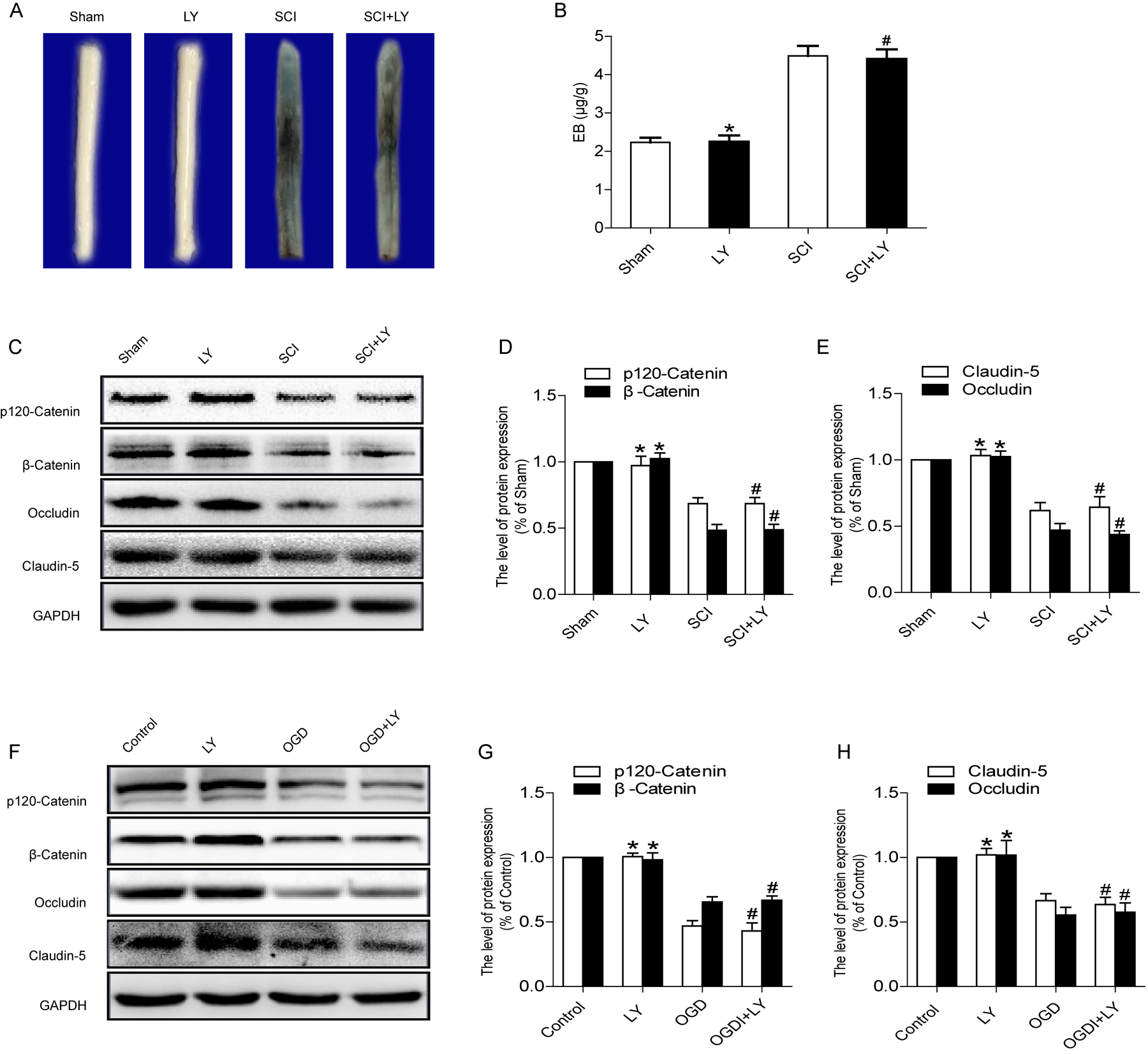

Supplement: Supplementary file 1 — Figure S1 LY294002 doesn't have a toxic and devastating effect by itself in the in vivo and in the in vitro treatments. (A) Representative whole spinal cords show that Evan's Blue dye permeabilized into injury spinal cord at 1 day (n = 4/group). (B) Quantification of the amount of Evan's Blue at 1 day (μg/g). (C) Protein expressions of p120‐Catenin, β‐Catenin, Occludin, Claudin‐5 in the spinal cord segment at the contusion epicentre. (D and E) The optical density analysis of p120‐Catenin, β‐Catenin, Occludin and Claudin‐5 protein. (F) Protein expressions of p120‐Catenin, β‐Catenin, Occludin, Claudin‐5 in endothelial cells. (G and H) The optical density analysis of p120‐Catenin, β‐Catenin, Occludin and Claudin‐5 protein. *represents P > 0.05 versus the Sham group, #represents P > 0.05 versus the SCI group. [file JCMM-20-1062-s001.tif]
